# A Novel Variant of *OCT4* Entitled OCT4B3 is Expressed in Human Bladder Cancer and Astrocytoma Cell Lines

**Published:** 2017

**Authors:** Ensieh M. Poursani, Majid Mehravar, Bahram Mohammad Soltani, Seyed Javad Mowla, James E. Trosko

**Affiliations:** 1. Department of Molecular Genetics, Faculty of Biological Sciences, Tarbiat Modares University, Tehran, Iran; 2. Food Safety Toxicology Center, Department of Pediatrics and Human Development, Michigan State University, East Lansing, Michigan (MI), USA

**Keywords:** Alternative splicing, Cell lines, Gene, Stem cells

## Abstract

**Background:**

Alternative splicing is an important mechanism that regulates gene expression and function in human cells. *OCT4*, a crucial pluripotency marker in embryonic stem/carcinoma cells generates several spliced variants in different cell types and cancers. The expression of *OCT4* in cancers has been challenged in many studies. The existence of several *OCT4* spliced variants and absence of specific discriminating primers is the main reason of this controversy. Therefore, using specific primers and discriminating *OCT4* variants from each other might help to reduce these discrepancies in carcinogenesis and stem cell researches.

**Methods:**

17 various human cancer, pluripotent and normal cells were cultured and their RNAs were extracted. Related cDNAs were synthesized and the expression pattern of *OCT4* variants was investigated by RT-PCR assay. PCR products were cloned into pTZ57R/T vector and their authenticity was confirmed by DNA sequencing.

**Results:**

Expression pattern of *OCT4* variants (OCT4A, OCT4B and OCT4B1) was analyzed by RT-PCR assay and the authenticity of PCR products was confirmed by DNA sequencing. A novel spliced variant of *OCT4* was discovered and named as OCT4B3. This variant was very similar to OCT4B2 transcript except that 207-nt of exon 1b is lost. Moreover, the expression pattern of OCT4B3 variant was investigated in 17 human cell types, where its expression was only found in astrocytoma and bladder cancer cell types 1321N1 and 5637, respectively.

**Conclusion:**

*OCT4* variants are differentially expressed in various human cancer cell lines. Moreover, a novel variant of *OCT4*, OCT4B3, was detected in two human cancer cell lines of bladder carcinoma (5637) and brain astrocytoma (1321N1) for the first time.

## Introduction

*OCT4* gene, an important stem cell marker, maintains stemness properties of Embryonic Stem (ES) and Embryonic Carcinoma (EC) cells ^1,2^. Up until now, it is known that *OCT4* pre-mRNA can produce various splice variants such as OCT4A, OCT4B, OCT4B1 and also OCT4B2 under different situations ^3,4^. The OCT4A isoform is known as a prominent factor that sustains self-renewal and pluripotency in ES and EC cells. Compared to OCT4A, OCT4B isoforms such as OCT4B-164, OCT4B-190 and OCT4B-265 cannot sustain stemness properties in mentioned cells and respond to the cell stresses. OCT4B1 is a newly discovered variant and is known to be expressed in undifferentiated stem cells and down-regulated during induction of differentiation ^4^. The existence of several *OCT4* spliced variants and 7 transcribed pseudogenes might interfere in detection and discrimination of OCT4A, the main pluripotency marker, from other *OCT4* gene products (spliced variants and pseudogenes) ^5^. The alternative splicing is considered as a major mechanism for gene expression and function regulation and a source of protein diversity which resulted in expanding protein function repertoire in mammals ^6–8^. *OCT4* gene can be influenced by alternative splicing and generates multiple spliced variants under different conditions and/or tissues. Therefore, this mechanism of gene regulation might play a critical role in orchestrating complex regulatory functions within *OCT4* gene.

In this study, specific primers located in different sites of the *OCT4* gene were used and the expression pattern of *OCT4* variants investigated by RT-PCR and sequencing approaches.

## Materials and Methods

### Cell culture

Various human cell lines were provided by Pasteur Institute, and Avicenna Research Institute of Iran, Tehran. The human cell lines of T-cell lymphoma (Jurkat), Burkitt’s lymphoma (Raji), ovary adenocarcinoma (Ovcar3), glioblastoma (U87), urinary bladder carcinoma (5637), and pluripotent embryonic carcinoma (NCCIT) were cultured in RPMI-1640 medium supplemented with 10% FBS, 100 *mM* sodium pyruvate, penicillin (100 *u/ml*) and streptomycin (100 *µg/ml*). Cell types of breast adenocarcinoma (MCF-7), pluripotent embryonic carcinoma (NT2), cervix adenocarcinoma (HeLa), glioblastoma (A172), medulloblastoma (Daoy), embryonic kidney (HEK-293), hepatocellular carcinoma (HepG2), human brain astrocytoma (1321N1) and bone marrow normal fibroblast (HS5) were cultured in High Glucose Dulbecco’s Modified Eagle Medium (DMEM, 4500 *mg/l*) supplemented with 10% FBS and sodium pyruvate and penicillin/streptomycin as described above. Y-79 cell line was cultured in RPMI-1640 medium supplemented with 20% FBS.

### RNA extraction and cDNA synthesis

RNA extraction was performed using TRIzol reagent (Invitrogen, UK) according to the instruction of manufacturer. Quantity and quality of isolated RNAs were evaluated by spectrometry and electrophoresis on the 1% agarose gel. Total RNAs were digested with RNase-free DNaseI (Fermentase, Lithuania) to remove any unwanted DNA contamination. The first strand of cDNAs was synthesized using 2 *µg* of each DNase-treated RNA, RT enzyme (Fermentase, Lithuania) and oligo-dT primer according to the manufacturer’s instruction. GAPDH was used as an internal control to assess quality of synthesized cDNAs. The efficiency of DNase treatment and lack of DNA contamination was tested by having No-RT controls.

### Reverse Transcription-Polymerase Chain Reaction (RT-PCR)

RT-PCR was performed with the red master mix (Amplicon) according to the instruction of manufacturer. Primer sets of AF/RB1, FB/RB5, FB/RB4, B2S/RB2 and GAPF/GAPR were used to amplify OCT4A, OCT4B, OCT4B1, OCT4B3, and GAPDH, respectively. The sequences of used primers were as follow: B2S, (5′-AGGGCTCTTTGTCCACTTTGTATAG-3′); RB2, (5′-CTCAAAGCGGCAGATGGTCG-3′); AF,5′-CTTC TCGCCCCCTCCAGGT-3′; RB1,5′-AAATAGAACCC CCAGGGTGAGC-3′; RB4,5′-CCCCCTGTCCCCCAT TCCTA-3′; RB5,5′-GGCTGAATACCTTCCCAAATA GA-3′; GAPF(5′-GCCACATCGCTCAGACAC-3′) and GAPR (5′-GGCAACAATA TCCACTTTACCAG-3′).

RT-PCR approach was performed using 0.5 *µl* of cDNA and No-RT samples (samples without RT-enzyme that are used as a negative controls to find out any possible DNA contamination) and 4 *pmol* of mixed Forward and Reverse primers in total vol. of 10 *µl*. Polymerase chain reaction for OCT4A, OCT4B, OCT4B1 and OCT4B3 transcripts was performed under the following cycling conditions: initiation at 94*°C* for 4 *min*, amplification for 35 cycles with denaturation at 94*°C* for 30 *s*, annealing at 65*°C* for 30 *s* and extension at 72*°C* for 30 *s*, with a final extension at 72*°C* for 7 *min*. The thermal profile for GAPDH was performed for 28 cycles with annealing at 58*°C* for 30 *s* and extension at 72*°C* for 15 *s*.

The amplicon sizes were 495 *bp*, 245 *bp*, 492 *bp*, 511 *bp* and 140 *bp* for OCT4A, OCT4B, OCT4B1, OCT4B3 and GAPDH, respectively. Consequently, PCR products were confirmed by DNA sequencing.

### DNA cloning and sequencing

511-*bp* PCR products were excited from agarose gel, purified by DNA purification kit (GeneAll Biotechnology, South Korea) and cloned into the pTZ57R/T vector. Positive colonies containing recombinant vectors were selected by colony PCR and were cultured in the liquid LB media containing ampicillin antibiotic for an overnight. Recombinant vectors were extracted by plasmid extraction kit (GeneAll Biotechnology, South Korea) and sequenced (Applied Biosystems, South Korea).

## Results

### OCT4 variants are differentially expressed in various cancer cell lines

**Figure 1.**
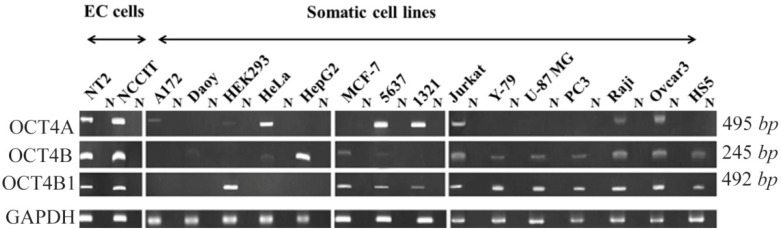
RT-PCR analysis of OCT variants in various human cell lines. The expression pattern of *OCT4A*, *OCT4*B and *OCT4*B1 variants were investigated by primer sets of AF/RB1, FB/RB5 and FB/RB4, respectively. The predicted amplicon sizes were 495 *bp* for *OCT4A*, 245 *bp* for *OCT4*B, 492 *bp* for *OCT4*B1 and 140 *bp* for GAPDH transcripts. GAPDH was used as internal control for each sample. N; No-RT negative controls for each sample.

The expression pattern of OCT4A, OCT4B and OCT4B1 was examined by RT-PCR assay using primer sets of AF/RB1, FB/RB5 and FB/RB4, respectively. Our data revealed that OCT4A is expressed in NT2, NCCIT, HeLa, 5637, 1321N1, Jurkat, Raji, Ovcar3, A172 and HEK293 cell lines. The OCT4B transcript was detected in NT2, NCCIT, HepG2, MCF7, Jurkat, Y-79, U-87 MG, PC3, Raji, Ovcar3, HS5, HeLa and 5637 cell lines. Cell lines of NT2, NCCIT, MCF7, 5637, 1321N1, Jurkat, Y-79, U-87 MG, PC3, Raji, Ovcar3 and HS5 expressed OCT4B1 at transcript level (Figure 1).

### Identification of a novel OCT4 spliced variant, OCT4B3

**Figure 2.**
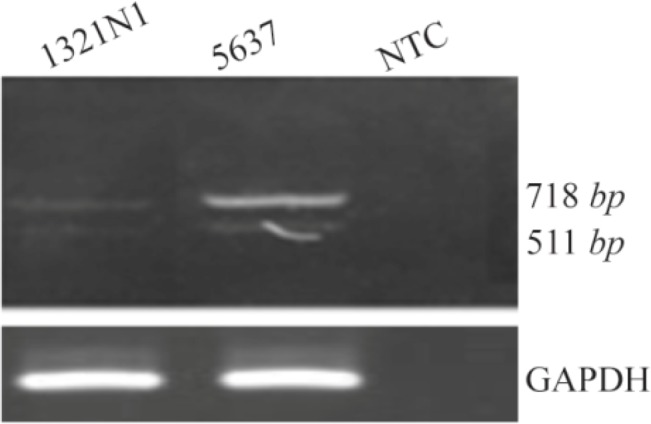
A) RT-PCR analysis of OCT4B3. Using primer set B2S/RB2 we detected a 511 bp PCR product in the 5637 and 1321 cell lines which was corresponded to the OCT4B3 transcript. The 718 *bp* fragment was considered to be the OCT4B2 variant. GAPDH was used as internal control for RT-PCR and NTC is non-template control.

During expression analysis of *OCT4* variants, specifically OCT4B2, new transcript nearly 207-nt shorter than OCT4B2 was detected using B2S/RB2 primer set (Figure 2). Performing PCR with nested primers did not eliminate this extra PCR product. Therefore, this fragment was cloned into T/A cloning vector (PTZ-57R/T) and sequenced. The sequencing results were analyzed using Chromas software and Blast in NCBI database. Finally, it was found that *OCT4* generates a novel spliced variant. This new *OCT4* transcript was registered in GenBank and named OCT4B3 (GenBank accession number; KJ624996).

### OCT4B3 is generated by alternative splicing of OCT4B2

**Figure 3.**
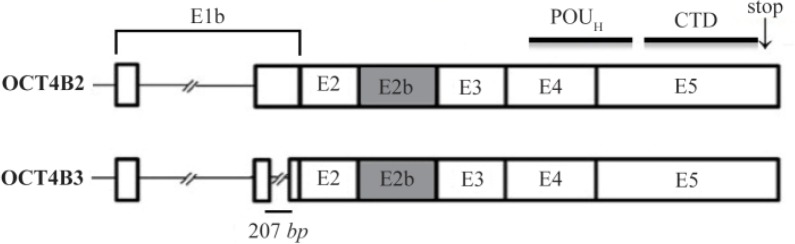
Schematic structure of OCT4B3 transcript and protein. This variant is composed of 6 exons (E1b, E2, E2b, E3, E4 and E5) and is very similar to the OCT4B2 transcript, however, it is lost a 207-nt fragment of the Exon1b. The OCT4B3 transcript can produce a hypothetical protein includes 164 residues that is the same with OCT4B-164 protein and composed of POU_H_ and C-Terminal domains

Bioinformatic and BLAST analysis revealed that OCT4B3 is very similar to the OCT4B2 transcript. OCT4B3 is composed of exons of 1b, 2, 2b, 3, 4 and exon5, the same as OCT4B2, but it lost 207 nucleotides of exon 1b compared to the OCT4B2 transcript (Figure 3). It was supposed that OCT4B2 mRNA might probably splice and lose 207 nucleotide of the exon 1b and as a result generates the OCT4B3 transcript.

### OCT4B3 is expressed in human brain astrocytoma and bladder carcinoma cell lines

**Figure 4.**
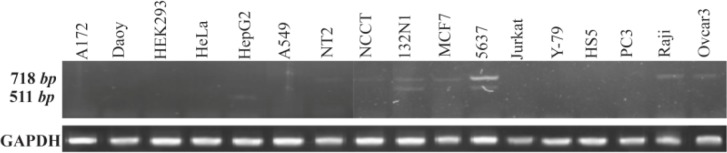
Expression pattern analysis of OCT4B3 by RT-PCR. Using primer set B2S/RB2, the expression pattern of OCT4B3 was investigated in various human cell lines. 718 *bp* and 511 *bp* PCR products considered to be OCT4B2 and OCT4B3, respectively. Among 17 cell lines, OCT4B3 was detected just in 1321N1 and 5637 cell lines. GAPDH was used as internal control of RT-PCR with the size of 140 *bp.*

The expression pattern of OCT4B3 was evaluated in various human cell lines with RT-PCR. Among 17 human different cell types (A172, Daoy, HEK293, HeLa, HepG2, MCF-7, NT2, 5637, 1321N1, NCCIT, Jurkat, Y79, HS5, PC3, Raji, OVCAR3 and A549), OCT4B3 transcript was detected just in 1321N1 and 5637 cell lines (Figure 4).

## Discussion

Alternative splicing is an important mechanism which confers protein diversity in mammals especially in human. This process permits a single gene to produce several transcripts and proteins with different functions in various tissues and conditions. Recent genome-wide analysis suggests that nearly 95% of human genes are alternatively spliced ^9,10^. In many cases, alternative splicing patterns are tissue specific events ^11^. Therefore, regulatory elements in the pre-mRNA sequence (introns and exons) are associated with tissue specificity of these splice variants ^12^. *OCT4* gene can generate various isoforms by alternative splicing expressed in different tissues and have disparate functions ^13^. For example, OCT4A is an important transcription factor that donates stemness properties to the ES and EC cells ^14,15^. OCT4B-190 and OCT4B-265 are two other *OCT4* spliced variants that response to cell stress ^16,17^. OCT4B1 is expressed in the stem cells and undifferentiated cells and suggests to be a stemness marker. OCT4B2 is expressed in different cancer cells and up-regulated under stress conditions, as well ^4,18^. Here, the expression pattern of known *OCT4* variants was investigated (OCT4A, OCT4B and OCT4B1) by RT-PCR assay. Our results indicated that OCT4A is expressed in pluripotent cells such as embryonic carcinoma cells (NT2 and NCCIT) and some other cancer cell lines. OCT4B and OCT4B1 transcripts were detected in the most of examined cells in this study.

Moreover, another *OCT4* spliced variant was introduced, OCT4B3 that is very similar to the OCT4B2 transcript which lacks 207-nt of exon 1b. This variant has a low expression as usual, and was detected in the human bladder cancer (5637) and astrocytoma (1321N1) cell lines. It was supposed that OCT4B2 might lose a 207-nt fragment of its Exon1b by splicing and generates OCT4B3 under special conditions. However, it needs more experimental evidences to confirm this hypothesis definitely. Bioinformatic analysis revealed that OCT4B3 transcript can produce a hypothetical protein which is the same with OCT4B-164 isoform and composed of POU_H_ and N-Terminal (NTD) domains (Figure 3). Since cancer cell lines are a mixture of “cancer stem cells” and “cancer cells”, reflecting the complex mixture of these cancer stem cells, stromal cells and invasive immune cells in an *in vivo* tumor, internal special micro-environmental conditions affect a complex interaction of these cell types that induce the production of these various spliced variants.

## Conclusion

Using specific primers, we have discovered a novel variant of *OCT4*, named as OCT4B3. The sequence of the variant is very similar to OCT4B2 transcript, except that a 207-nt segment of exon 1b is lost in OCT4B3. The expression of the variant was detected in two human cancer cell lines of bladder carcinoma (5637) and brain astrocytoma (1321N1) cells.
